# Supplementary material for: Alteration of Mitochondrial Integrity as Upstream Event in the Pathophysiology of SOD1-ALS
Source: Cells. 2022 Apr 6;11(7):1246. doi: 10.3390/cells11071246 (PMC8997900; doi:10.3390/cells11071246)
Supplement: Supplementary file 1 [file cells-11-01246-s001.zip › cells-1624641-supplementary.pdf]

Supplements

Figure S1

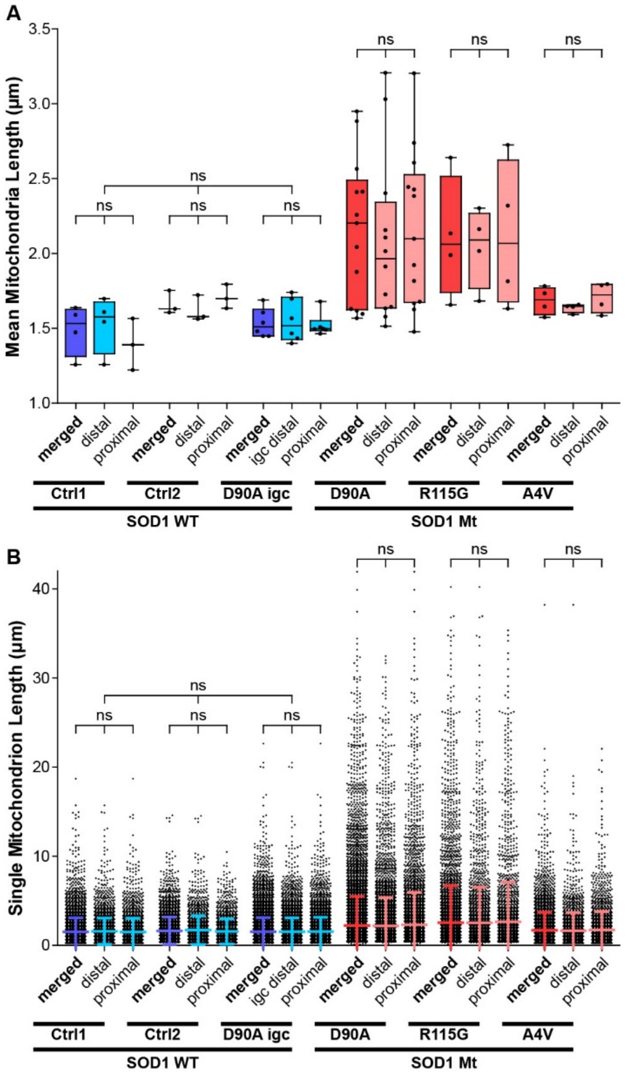

Figure S1. Increased elongation of mitochondria in mutant SOD1 spinal MNs. Shows all single iPSC lines corresponding to fig. 4. Validates (i) the merging of distal and proximal data points and (ii) the pooling of SOD1 WT and Mt lines in fig. 3c, d, f. Ns: no significant change in any pairwise comparison by one-way ANOVA with Bonferroni post-hoc test. (A, B) Note, distal and proximal data points were indistinguishable for each line, thereby validating their merging for fig. 3c, d, f. Moreover, all three SOD1 WT lines (Ctrl1, Ctrl2, D90A igc) were indistinguishable from each other, thereby validating their pooling in fig. 3c, d, f. (A) Box plots of mean mitochondria length values per experiment (black dots) for each respective line with 25-75% interquartile range (box), median (horizontal line) and non-outlier range (1.5-fold interquartile range added above and below to box, whiskers). (B) Length distribution of single mitochondria displayed as scatter dot plot (i.e. each dot presents one mitochondrion).

Figure S2

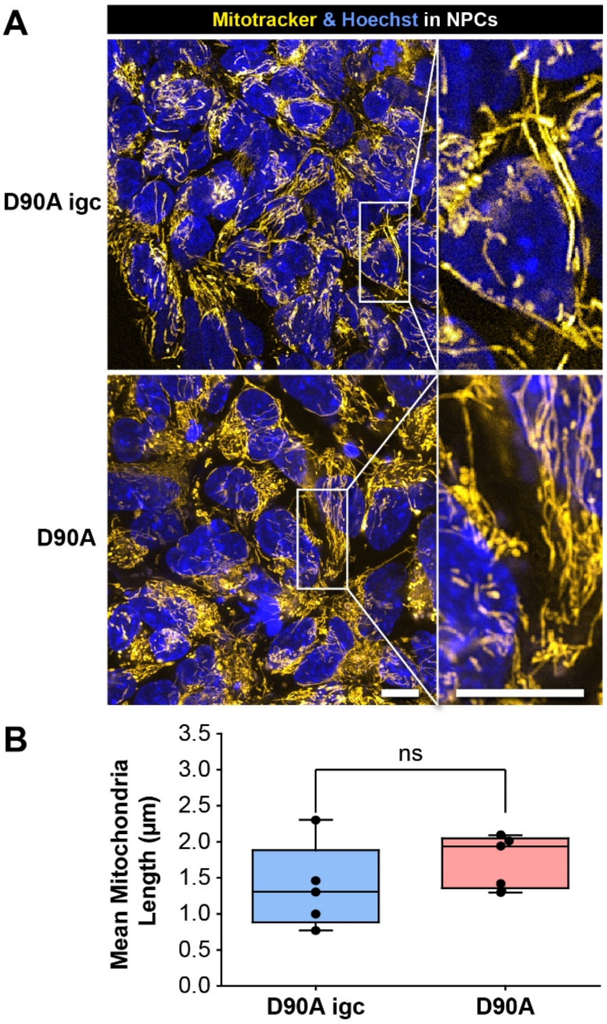

Figure S2. SOD mutant neuronal progenitor cells (NPCs) did not exhibit increased mitochondria elongation prior to differentiation to spinal MNs.

(A) Representative fluorescence images of SOD Wt (D90 igc) and SOD Mt (D90A) NPCs during expansion (as illustrated in fig. 4a) stained live with mitotracker and Hoechst revealed no apparent difference in mitochondria length. Scale bars = 10 µm, boxed areas are magnified on the right. **(B)** Quantification of (A), box plots of mean mitochondria length values per experiment (N = 5, black dots) for each respective line with 25-75% interquartile range (box), median (horizontal line) and non-outlier range (1.5-fold interquartile range added above and below to box, whiskers). ns not significant according to unpaired two-tailed Student's t-test.

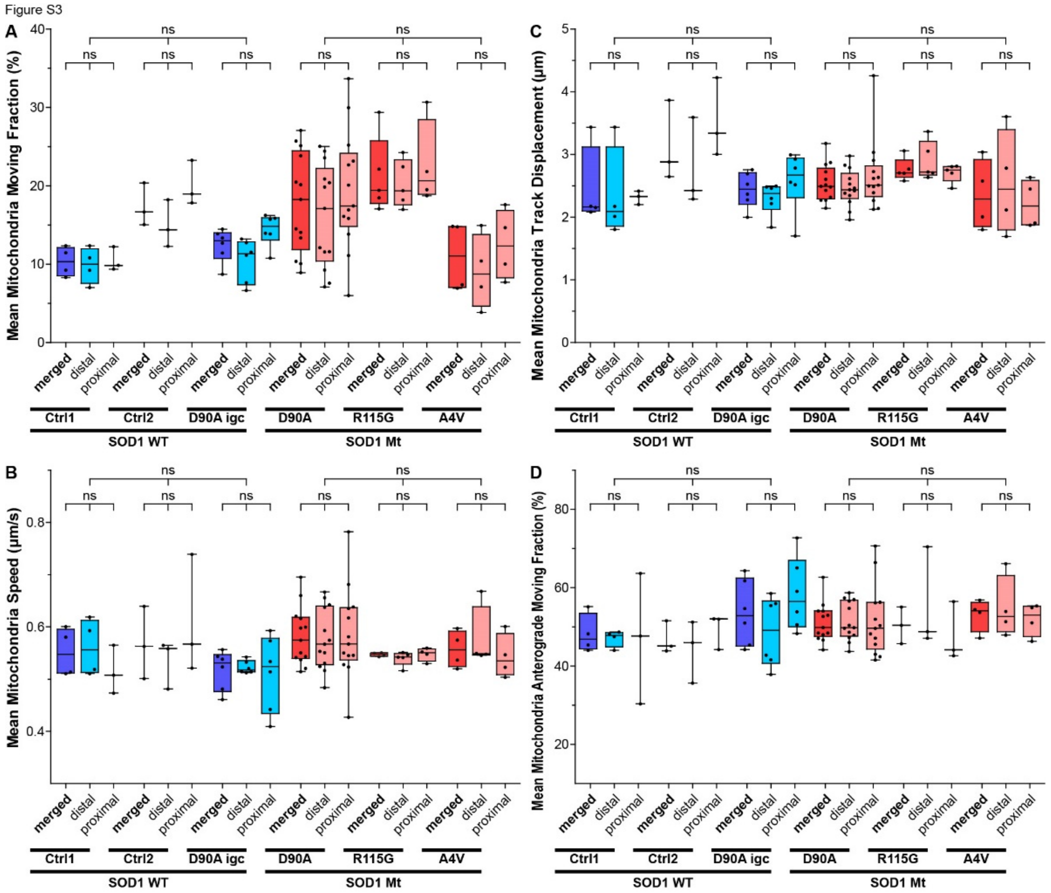

Figure S3. Altered motility of mitochondria in mutant SOD1 spinal MNs.

Shows all single iPSC lines corresponding to fig. 5. Validates (i) the merging of distal and proximal data points and (ii) the pooling of SOD1 WT and Mt lines in fig. 4a-f. Ns: no significant change in any pairwise comparison by one-way ANOVA with Bonferroni post-hoc test. **(A-D)** Note, distal and proximal data points were indistinguishable for each line, thereby validating their merging for fig. 4a-f. Moreover, all three SOD1 WT lines (Ctrl1, Ctrl2, D90A igc) and all three SOD1 Mt lines (D90A, R115G, A4V) were indistinguishable from each other, thereby validating their respective pooling in fig. 4a-f. All box plots are showing mean values per experiment (black dots) for each respective line with 25-75% interquartile range (box), median (horizontal line) and non-outlier range (1.5-fold interquartile range added above and below to box, whiskers).

Figure S4

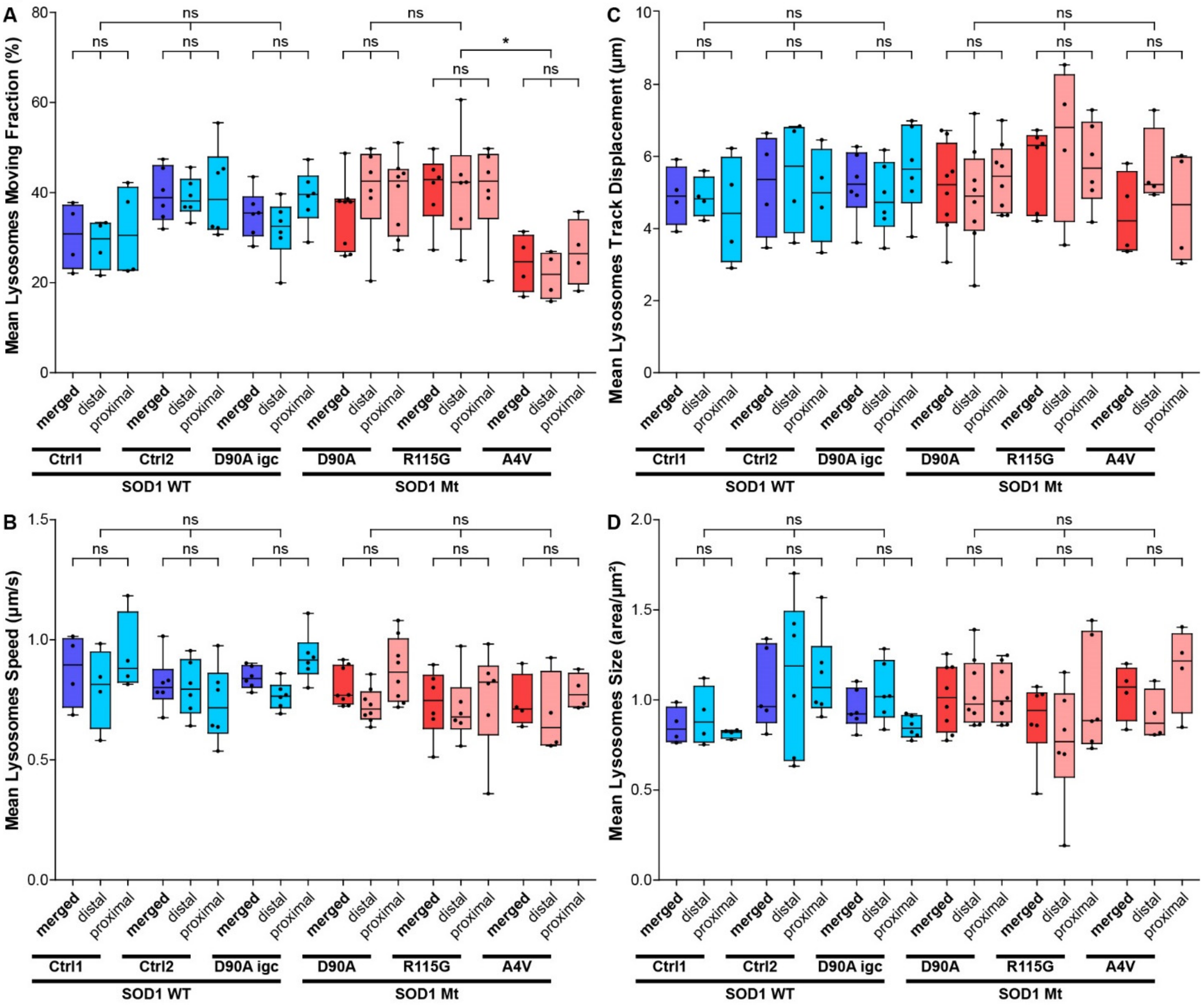

Figure S4. Lysosomal size and motility was not altered in mutant SOD1 spinal MNs.

Shows all single iPSC lines corresponding to fig. 6. Validates (i) the merging of distal and proximal data points and (ii) the pooling of SOD1 WT and Mt lines in fig. 5a-d. Asterisks: highly significant increase in respective pairwise comparison, one-way ANOVA with Bonferroni post test. * $p<0.05$, ** $p<0.01$, *** $p<0.001$, **** $p<0.0001$, ns not significant. **(A-D)** Note, distal and proximal data points were indistinguishable for each line, thereby validating their merging for fig. 5a-d. Moreover, all three SOD1 WT lines (Ctrl1, Ctrl2, D90A igc) and all three SOD1 Mt lines (D90A, R115G, A4V) were indistinguishable from each other (exception: panel A, line A4V), thereby validating their respective pooling in fig. 5a-d. All box plots are showing mean values per experiment (black dots) for each respective line with 25-75% interquartile range (box), median (horizontal line) and non-outlier range (1.5-fold interquartile range added above and below to box, whiskers).

Figure S5

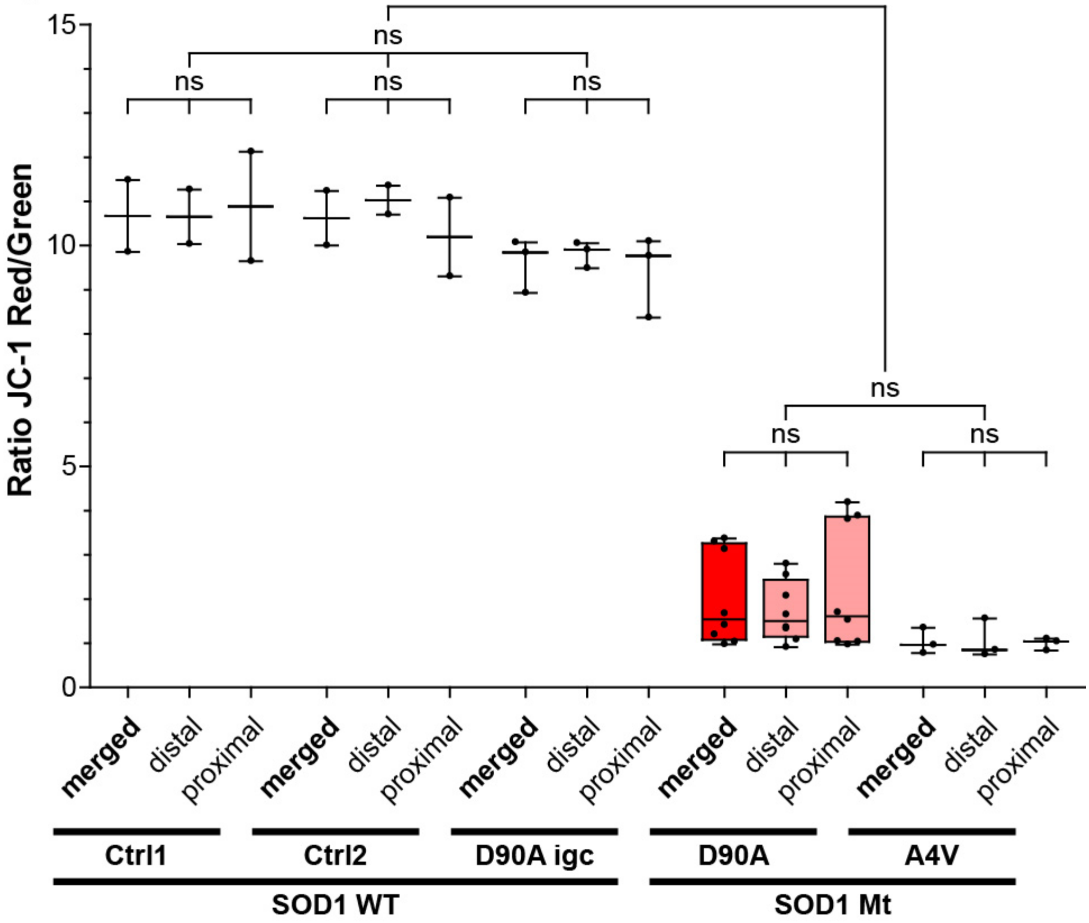

Figure S5: Reduced mitochondrial inner membrane potential in mutant SOD1 spinal MNs.

Shows all single iPSC lines corresponding to fig. 7. Validates (i) the merging of distal and proximal data points and (ii) the pooling of SOD1 WT and Mt lines in fig. 6a, b. Asterisks: highly significant increase in respective pairwise comparison, one-way ANOVA with Bonferroni post test. * $p<0.05$, ** $p<0.01$, *** $p<0.001$, **** $p<0.0001$, ns not significant. **(A, B)** Note, distal and proximal data points were indistinguishable for each line, thereby validating their merging for fig. 6a, b. Moreover, all three SOD1 WT lines (Ctrl1, Ctrl2, D90A igc) and all three SOD1 Mt lines (D90A, R115G, A4V) were indistinguishable from each other, thereby validating their respective pooling in fig. 6a, b. All box plots are showing mean values per experiment (black dots) for each respective line with 25-75% interquartile range (box), median (horizontal line) and non-outlier range (1.5-fold interquartile range added above and below to box, whiskers).
